# Information flow in cultured neuronal networks by transfer entropy

**DOI:** 10.1016/j.bpr.2026.100278

**Published:** 2026-07-20

**Authors:** Wataru Minoshima, Yumi Segawa, Chie Hosokawa

**Affiliations:** 1 Department of Chemistry, Graduate School of Science, Osaka Metropolitan University, Osaka, Japan; 2 Advanced ICT Research Institute (Kobe), National Institute of Information and Communications Technology, Kobe, Japan

## Abstract

Neurons form functional connections in neuronal networks of the brain. Neurotransmission refers to the information flow of electrical signals between neurons, and changes in the strength of this flow characterize brain functions. An evaluation method for neuronal information flow strength is necessary to elucidate the basic principles of brain function. To analyze the strength of information flow, identifying information transmission between time series of electrical spikes in neuronal networks, such as spike trains, is required. In this study, we evaluated the information flow strength in neuronal networks using transfer entropy (TE), an analysis method based on information theory, to elucidate causal relationships between two spike trains. Cultured rat hippocampal neuronal networks were used as living brain models, and extracellular monitoring of action potentials (expressed as spikes) was performed using microelectrode arrays. Spike trains of spontaneous activity and stimulus-evoked responses were measured in multiple neurons, and the causal relationship between electrode pairs was evaluated as an index of information flow between neurons using TE. From the results of spontaneous activity and evoked responses, it was suggested that TE values between electrodes increased with culture days and that the information flow strength was enhanced by network maturation. Furthermore, the correlation between TE values and neuronal population distance was evaluated using TE analysis. We found that neurons that received strong inputs slightly overlapped in their spontaneous activity and evoked responses. These findings suggest that spontaneous activity and evoked responses exhibit similar patterns in neuronal networks. In the graph theoretical analysis based on TE values, the network topology exhibited changes that strengthened information flow over 70 days in culture. Therefore, TE analysis is an effective tool for estimating the information flow between neurons based on neuronal electrical activity recorded at multiple sites.

## Why it matters

The spatial propagation of electrical activity in neuronal networks plays an important role in brain function. To understand the spatiotemporal dynamics of neuronal networks, it is necessary to elucidate the spatial information flow between neurons. In this study, we analyzed the information flow strength using transfer entropy (TE) from the electrical activity of rat hippocampal neurons cultured on microelectrode arrays. Using TE analysis, we found an increase in information flow with network maturation, a relationship between information flow strength and neuronal population distance, and a pattern similarity between spontaneous activity and evoked responses induced by electrical stimulation. These findings indicate that TE analysis is a promising method for evaluating information flow in neuronal networks.

## Introduction

In the brain, neurons are connected via synapses to form complex neuronal networks. The spatial propagation of neuronal electrical activity generates spatial and temporal patterns related to brain function. Recent studies have discussed temporal neuronal electrical activity patterns using multi-neuron activity recordings, such as changes in synaptic connections owing to electrical stimulation,^1^^,^^2^^,^^3^^,^^4^^,^^5^ pattern modification during the maturation of neuronal networks,^6^^,^^7^^,^^8^^,^^9^ and synchronized activity.^10^^,^^11^^,^^12^^,^^13^ In contrast, spatial activity patterns are modified by changes in the information flow between neurons. Methods for evaluating the spatial distribution of information flow strength between neurons in neuronal networks during network maturation are required to understand brain function.

Microelectrode arrays (MEAs) enable long-term and noninvasive extracellular recordings from large numbers of neurons in a stable manner.^1^^,^^6^^,^^10^^,^^11^^,^^14^^,^^15^^,^^16^ In conventional MEAs, more than 60 electrodes are fabricated on the substrate surface. Extracellular potential measurements have been used to evaluate neuronal activity, such as spontaneous activity patterns and synchronization of neuronal electrical activity, which indicate changes in the connectivity of multiple neurons as the neuronal network matures.^1^^,^^10^^,^^17^ Furthermore, alterations in neuronal connectivity induced by external inputs can be evaluated from the responses evoked by current stimulation. However, previous studies have mainly focused on the temporal dynamics of neuronal electrical activity. Therefore, methods to evaluate the spatial information flow strength between neurons altered by effective neuronal connectivity in MEAs are required.

To estimate the information flow strength between neurons, it is necessary to analyze the electrical activity of neurons at multiple locations. Because the two neurons form excitatory connections through synapses, their spike timing is correlated. Generally, cross-correlation (CC) analysis is used to elucidate the temporal correlation between two spike trains.^18^ The values obtained by applying the CC indicate the temporal similarity between two spike trains; therefore, this method has been applied to multi-site recorded spike data to elucidate the synchronization of neuronal networks.^10^^,^^19^^,^^20^^,^^21^^,^^22^ However, several false-positive correlations have been detected using the CC in the case of high-frequency firing in neuronal networks.^23^ In contrast, information theory-based analysis is a promising tool for estimating the information flow strength between neurons.

In this study, we applied transfer entropy (TE) to estimate the information flow between neurons.^24^^,^^25^^,^^26^^,^^27^^,^^28^ In TE analysis, causal relationships can be evaluated from two different time-series signals, and this method can evaluate the direction of information flow.^24^ In addition, model-based studies and experimental results have reported that TE is expected to have high performance in elucidating the information flow strength from neuronal spike trains compared to CC analysis.^29^^,^^30^ We analyzed the changes in information flow strength in multi-site recorded spike trains in cultured rat hippocampal networks on MEAs using TE analysis. The changes in information flow strength from spontaneous activity and electrical responses evoked by electrical inputs as neuronal networks matured were evaluated, and the relationship between spontaneous activity and evoked responses in neuronal networks was discussed.

## Materials and methods

### Cultured rat hippocampal neurons in MEA dishes

**Figure 1:**
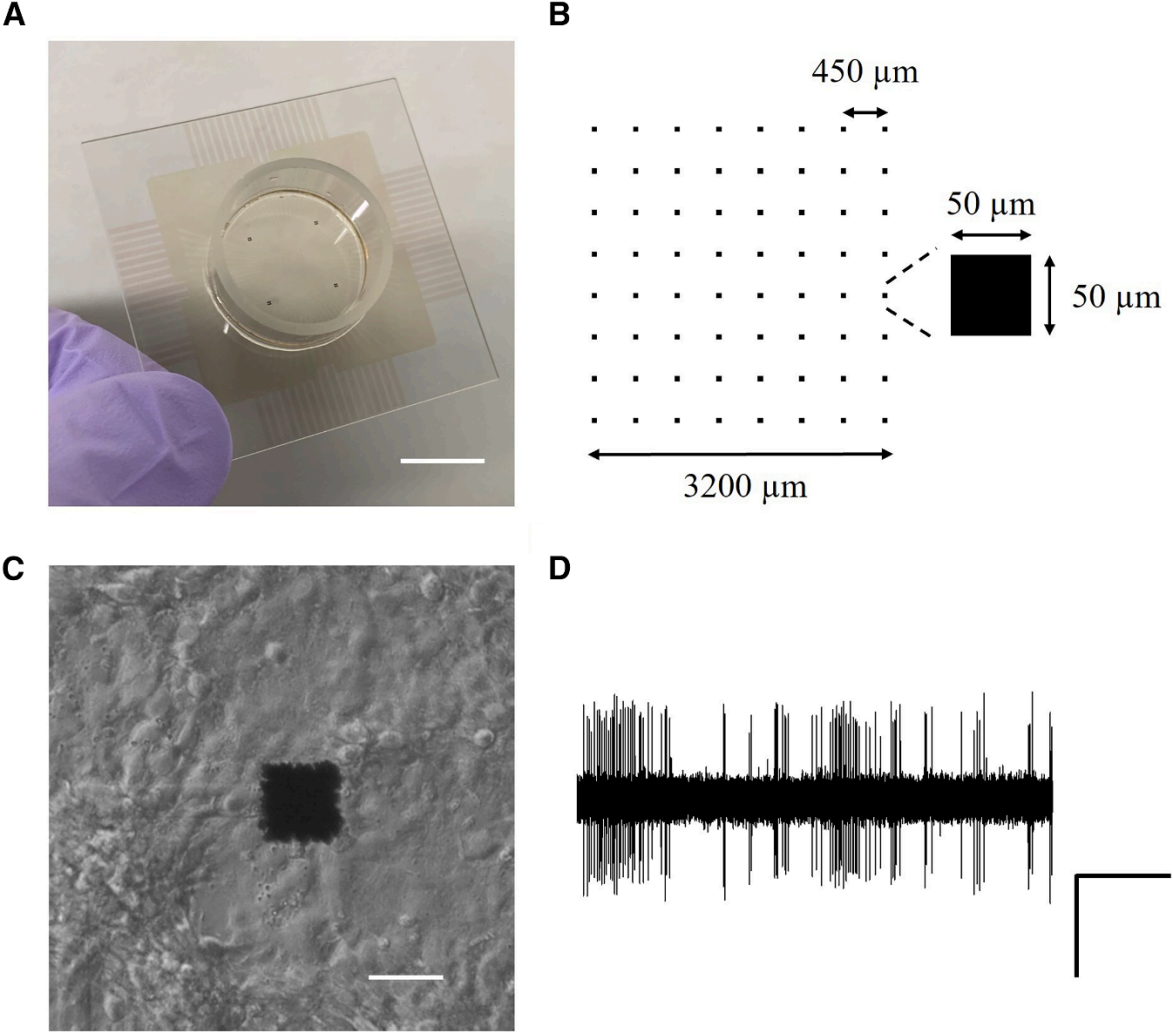
Experimental substrates and materials used in this study. (A) Photograph of MED probe. The scale bar corresponds to 10 mm. (B) Illustration of the microelectrode configurations on the MED probe. (C) Phase-contrast microscopy image of the cultured neuronal networks at 10 DIV on a MED probe. The black square is a microelectrode, and the scale bar corresponds to 50 μm. (D) Extracellular potentials of cultured neurons at 45 DIV on a single electrode. The horizontal and vertical scale bars correspond to 5 s and 50 μV, respectively.

All animal experimental procedures were performed under the approval of the Animal Experiment Management Committee of Osaka Metropolitan University. The animal experiments were conducted at room temperature. Hippocampal tissue from Wistar/ST rats (slc:Wistar/ST, Japan SLC) was removed from the fetal cerebrum. The tissue was treated with 0.063% trypsin (Thermo Fisher Scientific) dissolved in phosphate-buffered saline (Nissui Pharmaceutical) supplemented with 10 mM D-glucose at 37°C for 15 min. Cells were dissociated by mechanical pipetting, and the dissociated cells were seeded at a density of 2.6 × 10^5^ cells/cm^2^ in the center of the MEA culture dishes (MED probe, Alpha MED Scientific), as shown in Figure 1A. The 64 planar microelectrodes were arranged in an 8 × 8 array with 450 μm spacing in the MED probe, as shown in Figure 1B. The culture chamber of the MED probe was filled with Dulbecco’s modified Eagle’s medium: nutrient mixture F-12 (Thermo Fisher Scientific) mixed with a cocktail of 2.5 μg/mL insulin (Sigma-Aldrich), 1% penicillin-streptomycin (Thermo Fisher Scientific), 5% fetal bovine serum (FBS; Thermo Fisher Scientific), and 5% horse serum (HS; Thermo Fisher Scientific). FBS and HS were inactivated by incubation at 56°C for 30 min. The cultured cells were maintained for 24, 45, and 70 days after seeding in a CO_2_ incubator at 37°C with 5%/95% CO_2_/air saturation to form neuronal networks, as shown in Figure 1C. Half of the culture medium was replaced with fresh medium twice a week.

### Multi-site extracellular potential recording

Extracellular potentials were recorded using a MEA recording system (MED64, Alpha MED Scientific) containing a connector, amplifier, and a pair of analog to digital conversion boards (National Instruments). The measured signals were amplified 2,000 times and sampled at a frequency of 10 kHz. The analog signals were digitized with a 12-bit resolution. The MED64 was controlled using laboratory-made software based on LabVIEW 2011 (National Instruments), as shown in Figure 1D.

**Figure 2:**
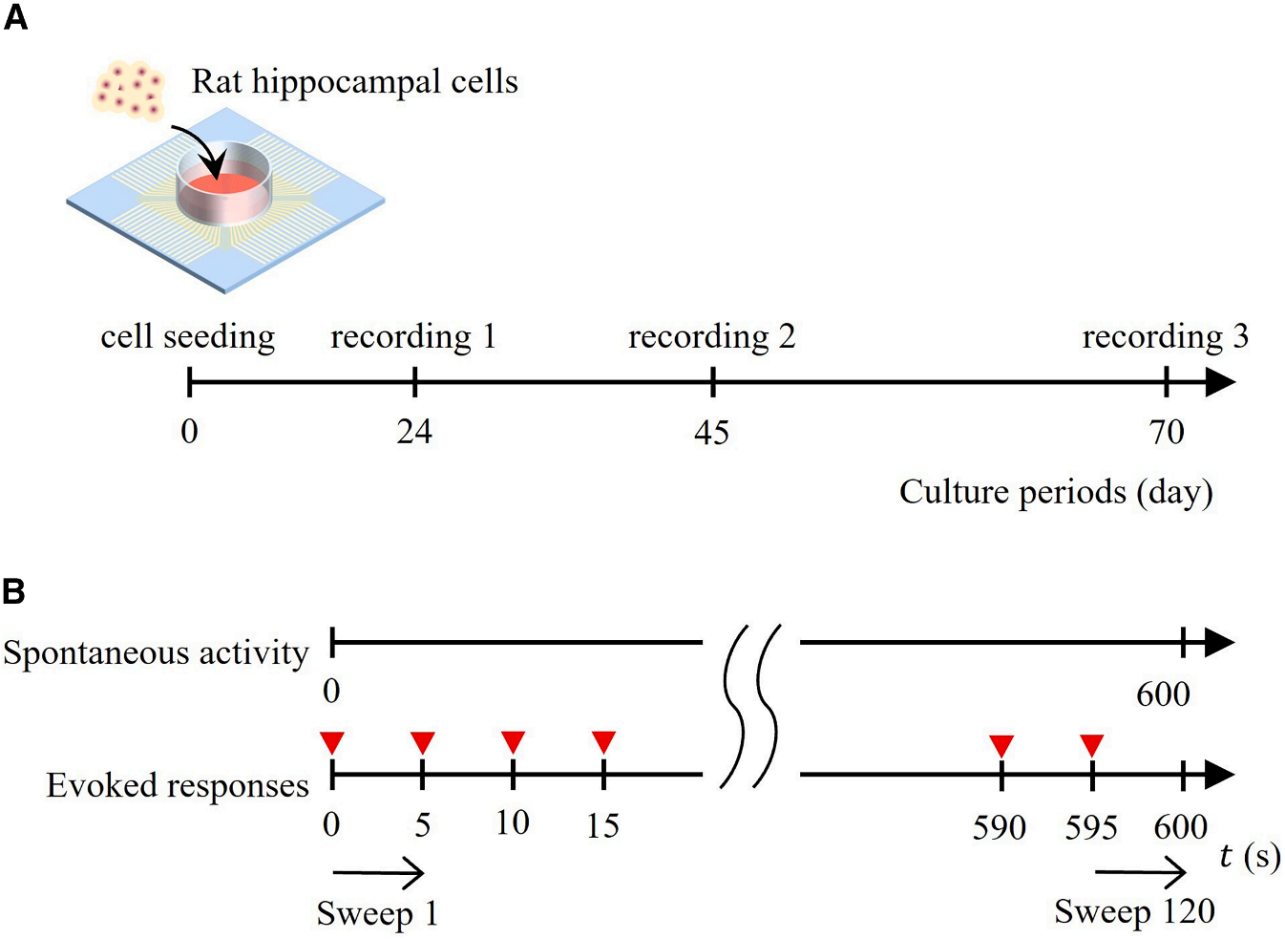
Schematic of the experiments for extracellular potential recording. (A) Experimental scheme for cell culture and measurement of the electrical activity of neurons during the culture period. (B) Schematic diagram showing the measurement of spontaneous activity and evoked responses. The red triangles indicate the time points of electrical input.

Spontaneous activity and evoked responses of neurons cultured in a MED probe at 24, 45, and 70 days *in vitro* (DIV) were measured for 600 s in each culture (Figure 2A). For the evoked response measurement, a biphasic current with an amplitude of 7.5 μA and a duration of 200 μs (100 μs in each phase) was applied from the amplifier to the stimulation electrodes every 5 s, as shown in Figure 2B. The stimulation sweep was defined as 5 s after each electrical stimulation, and spike trains in 120 sweeps were used for the analysis.

In this study, we maintained 11 cultures obtained from five pregnant rats (1–4 cultures per dam) and measured extracellular potentials longitudinally across 24, 45, and 70 DIV.

### Action potential spike detection

Spike detection was performed every 100 ms (1,000 digital data points) for each electrode. The spike detection procedure is described as follows. For spontaneous activity, the stored potential data were flattened by applying a 100–2,000 Hz band-pass third-order Butterworth filter, and the standard deviation (*σ*) of the noise amplitude was calculated. Peaks exceeding ±8*σ* were detected as spikes, representing action potentials. In the MEA measurements, the detected spikes are considered to originate from multiple neurons on a microelectrode. To analyze single-neuron properties, if two spikes were detected within 1 ms, the one with the smaller absolute amplitude was discarded to avoid the detection of multiple spikes. For peak detection, the “peak detector” function originally implemented in LabVIEW 2011 software was used.

To record the evoked responses in the experimental system, artifacts were observed at all recording sites immediately after the stimulation currents were applied. Therefore, the potential amplitudes for 5 ms (50 digital data points) after stimulation were shifted to 0, and spikes were detected in the same manner as the spontaneous activity.^14^^,^^31^^,^^32^ In the MED64 system used in this study, the circuit architecture of the amplifier employed for signal amplification required that the selected stimulation electrode be electrically switched from the recording electrodes. Consequently, the stimulation electrode could not be used to measure extracellular potentials, resulting in a total of 63 electrodes available for measurement. Spike detection was performed using a laboratory-made software based on LabVIEW 2011.

The firing properties of spontaneous activity in all 64 electrodes and the evoked responses in 63 electrodes were analyzed for each culture. The number of spikes per second summed over all 64 electrodes in a 600 s recording session was defined as the array-wide firing rate (AWFR). To evaluate stimulus-evoked activity, the time course of the total number of spikes in all 64 electrodes every 1 ms after stimulation was defined as the post-stimulus time histogram (PSTH). In this procedure, the PSTH values were averaged over 120 sweeps.

### TE analysis

**Figure 3:**
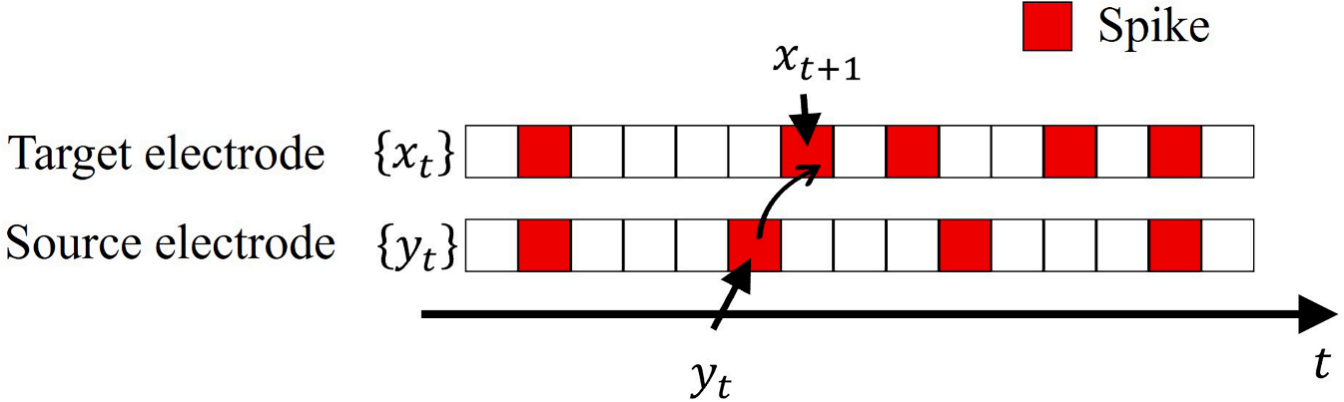
Schematic illustration of the TE calculation from two spike trains *x*_*t*_ and *y*_*t*_. The squares indicate a time bin of 1 ms. The red squares indicate the presence of one spike.

A 64-dimensional spike vector was created to evaluate the TE. The spike trains at the given electrodes were binned into 1 ms time windows, and each element in each time window was set to 1 and 0, indicating the presence and absence of spikes, respectively. Bin width was determined in accordance with a previous report.^29^ The spike vectors of electrodes *x* and *y* are defined as {*x*_*t*_} and {*y*_*t*_}, respectively, and the TE from electrode *y* to *x* (${TE}_{y\to x})$ is expressed as follows^33^^,^^34^:

(1) $${TE}_{y\to x}=\sum p\left(x_{t+1},x_{t},y_{t}\right)\log\frac{p\left(x_{t+1}|x_{t},y_{t}\right)}{p\left(x_{t+1}|x_{t}\right)}\text{,}$$

where *x*_*t*_ and *y*_*t*_ correspond to the spike status of target electrode *x* and source electrode *y*, respectively. *t* and *p*(*a*) correspond to the time point and event probability of spike generation at electrode *a*, respectively. The TE calculation procedure is shown in Figure 3. We applied logarithms with base 2, such that the units would be bits. The TE value is the Kullback-Leibler divergence; in short, ${TE}_{y\to x}\geq 0$ is guaranteed if there is information flow between given electrodes *x* and *y*, that is, if the past spike data for electrode *y* improve the prediction accuracy of spike generation at electrode *x*. In contrast, the TE value is 0 if there is no information flow between electrodes *x* and *y*. For the TE calculation, spike trains during the recording period of 600 s and between 5 and 50 ms after each stimulation (120 of the total number of stimuli) were used for spontaneous activity and evoked responses, respectively. To confirm that the TE values are not biased by the firing rate, we generated surrogate datasets in which the temporal structure of all recorded spike trains was randomized at 24, 45, and 70 DIV and calculated TE values from these surrogate spike datasets. The TE calculation procedure was performed using a laboratory-made software based on LabVIEW 2011.

The TE values were calculated from the spike vectors of the spontaneous activity and evoked responses of neurons cultured in a MED probe. These values were calculated for 4,032 pairs of electrodes, and a TE matrix was constructed. The total number of possible electrode pairs in the 64 electrodes was 4,096 (64 × 64), excluding the 64 same electrode pairs. Information flow is expected to generate between different electrodes, whereas no information flow is assumed to generate within the same electrode pairs (8 × 8). Therefore, we excluded the same electrode pairs because of a lack of information flow.

Furthermore, the relationship between the information flow strength and neuronal distance was analyzed. TE values were used as indicators of information flow strength, and electrode distance was used as an indicator of neuronal distance. Electrical stimuli were applied near the center of the MEAs, and the maximum distance between the recording sites and the stimulation sites was approximately 3,000 μm. The relationship between the TE value *x*_*i*_ and neuronal distance *y*_*i*_ was evaluated using Pearson’s correlation coefficient (PCC), which is presented as follows^35^:

(2) $$\text{PCC}=\frac{\sum \left(x_{i}-\bar{x}\right)\left(y_{i}-\bar{y}\right)}{\sqrt{\sum {\left(x_{i}-\bar{x}\right)}^{2}\sum {\left(y_{i}-\bar{y}\right)}^{2}}}\text{,}$$

where $\bar{x}$ and $\bar{y}$ are the average values of *x*_*i*_ and *y*_*i*_, respectively. PCC analysis was performed using Igor 6.3.

### TE-based graph theoretical analysis

To visualize the functional connectivity, we created the functional connectivity maps of spontaneous activity at 24, 45, and 70 DIV. The directed arrows from source electrodes to target electrodes were drawn for the subset of electrode pairs whose TE values exceeded the threshold defined by the mean + standard deviation in the TE matrix. Subsequently, we defined all drawn arrows as edges and regarded all electrodes connected by edges as nodes in the neuronal networks. We defined nodes that received highly dense inputs from other neurons as hubs. For a single electrode (node) in the MED probe, the maximum possible indegree is 63. In this study, we operationally defined hubs as nodes with an indegree exceeding approximately 25% of the theoretical maximum value. To account for variations in network activity, as all 63 electrodes were not constantly active across all experimental sessions, we established a practical hub-detection threshold of 15, which is closely aligned with 25% of the theoretical maximum possible indegree. Functional connectivity maps and graph theoretical analyses were performed using a custom script based on Python 3.11.

### Statistics

All statistical data were presented as mean ± standard error of the mean for 11 samples of cultured neurons. Pairwise comparisons among the three groups were performed using the Kruskal-Wallis test, followed by the Mann-Whitney *U* test. A pairwise Student’s *t* test was used to compare the summation of TE values between measured spontaneous activity and surrogate spontaneous activity on each culture day. Significance was defined when *p* values were less than 0.05. All statistical tests were performed using a custom-made script based on Python 3.13.

## Results

### Spike property changes under network maturation

**Figure 4:**
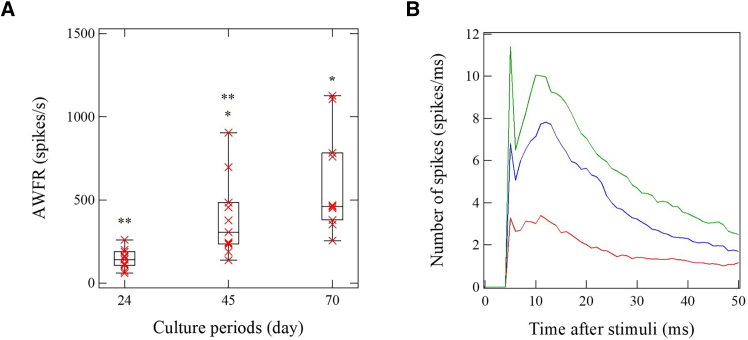
Firing properties of neuronal electrical activity at each culture stage. (A) AWFRs in each culture period. ∗*p* < 0.05 and ∗∗*p* < 0.01 indicate significant differences (Kruskal-Wallis test followed by Mann-Whitney *U* test). (B) PSTH of evoked responses in each culture period. The red, blue, and green lines correspond to 24, 45, and 70 DIV, respectively. The stimulation input was applied at 0 ms.

Spontaneous activity and evoked responses were measured in 11 cultures of MED probes at each culture period (24, 45, and 70 DIV), and firing properties were evaluated. The AWFRs are shown in Figure 4A. The mean values of the AWFR in the 11 experimental sessions were 143.2 ± 16.8, 388.4 ± 67.5, and 600.3 ± 86.3 spikes/s at 24, 45, and 70 DIV, respectively. The significance of the AWFR was confirmed at 24–45 DIV (*p* = 1.3 × 10^−3^, Kruskal-Wallis test followed by Mann-Whitney *U* test) and 45–70 DIV (*p* = 4.9 × 10^−2^, Kruskal-Wallis test followed by Mann-Whitney *U* test). The PSTH values for each culture period are shown in Figure 4B. The number of spikes in the evoked responses increased immediately after each stimulation and peaked 20 ms after the stimuli, and the firing rates of the evoked responses increased with the culture period. These results suggest that the ratio of spontaneous activity to evoked responses increased with culture days owing to network maturation, which is consistent with previous reports using MEAs.^9^^,^^11^^,^^13^^,^^36^^,^^37^

### Information flow in cultured neuronal networks using TE analysis

**Figure 5:**
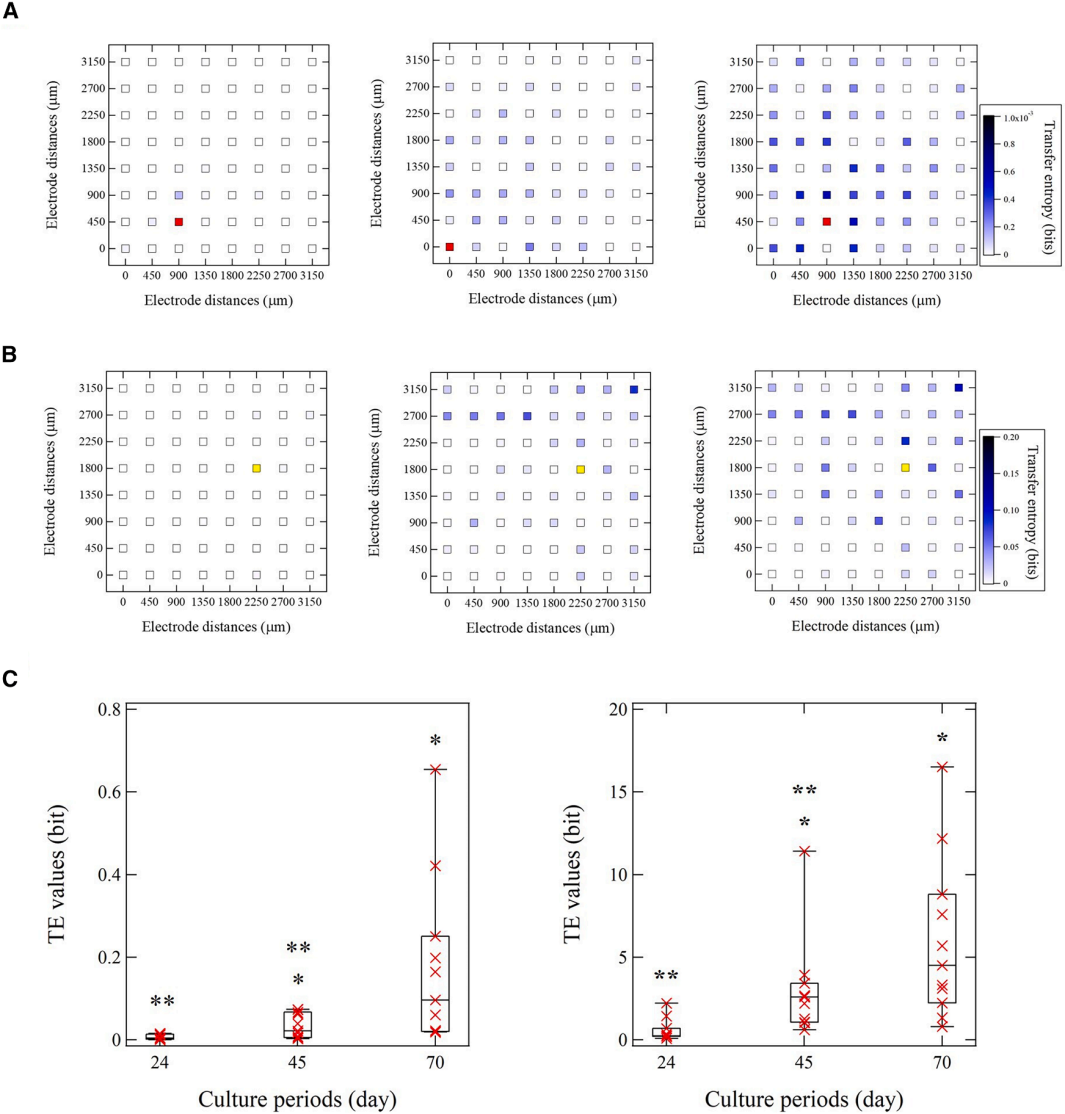
TE value distributions of all the electrodes in the MED probe. (A) Color map of TE values in each source electrode against the target electrode, which has the maximum TE value in spontaneous activity. The red squares indicate the electrodes with the maximum TE values. The culture periods were 24 (left), 45 (middle), and 70 (right) DIV, respectively. (B) Color map of the total TE values for each electrode against the stimulation site in stimulus-evoked responses. The yellow squares indicate the stimulation sites. The culture periods were 24 (left), 45 (middle), and 70 (right) DIV, respectively. (C) Summation of TE values for spontaneous activity (left) and stimulus-evoked responses (right). ∗*p* < 0.05 and ∗∗*p* < 0.01 indicate significant differences (Kruskal-Wallis test followed by Mann-Whitney *U* test).

To evaluate the spatial information flow between the electrodes, we analyzed the information flow in the selected electrodes for spontaneous and evoked responses. For spontaneous activity, we selected the electrode in which the maximum input was received as the maximum TE value. The TE values for each source electrode are presented in Figure 5. For each target electrode, the information flow strength to each target electrode was evaluated by summing the TE values from all 63 source electrodes, as shown in Figure 5A. The total TE values of spontaneous activity were 6.6 × 10^−3^ ± 1.7 × 10^−3^, 3.4 × 10^−2^ ± 8.6 × 10^−3^, and 0.18 ± 5.8 × 10^−2^ bit at 24, 45, and 70 DIV, respectively, as shown in Figure 5C (left). The significance of the TE values was confirmed at 24–45 (*p* = 8.6 × 10^−3^, Kruskal-Wallis test followed by Mann-Whitney *U* test) and 45–70 (*p* = 3.0 × 10^−2^, Kruskal-Wallis test followed by Mann-Whitney *U* test) DIV. The total TE values increased with the number of culture days, suggesting that effective neuronal connectivity was altered during network maturation, thereby increasing the strength of information flow between neurons.

We analyzed the 64-dimensional spike vector, which was set to 1 and 0, indicating the presence and absence of single action potentials, respectively. Considering the neuronal refractory period following an action potential at approximately 3 ms and synaptic delays at approximately 1 ms, a bin width of 1 ms for TE analysis is appropriate. Using a bin width smaller than 1 ms generates long sequences of zero-valued vectors, making the TE computation redundant. In contrast, a bin width larger than 1 ms increases the likelihood that spikes from nonsynchronized pairs of neurons will fall into the same bin. These results may be misinterpreted as synchronized spikes, thereby overestimating the TE values. To examine the effect of bin widths longer than 1 ms on TE values, we calculated the TE values of the spike data at 24 DIV with a 5 ms bin width that was longer than 1 ms. As shown in Figure S1, the summation TE values significantly increased with a bin width of 5 ms compared to a bin width of 1 ms (*p* = 5.6 × 10^−5^, Student’s *t* test), suggesting that the TE values were overestimated with a bin width larger than 1 ms. These results emphasize that a bin width of 1 ms is appropriate for TE analysis in cultured neuronal networks.

To facilitate a comparison with conventional methods for analyzing neuronal dynamics, the pairwise CC functions for all electrode pairs were calculated, and the coincident index (CI), as the synchrony index of neuronal networks, was evaluated. As shown in Figure S2, the summation of the CI values derived from the CC-based analysis did not increase significantly between 24 and 45 DIV and between 45 and 70 DIV.

In the electrical stimulus-evoked responses, we analyzed these values for 3,844 of 4,096 electrode pairs, excluding the same electrodes and stimulation sites. The total TE value for each target electrode at the stimulation site was obtained, as shown in Figure 5B. As shown in Figure 5C (right), the total TE values of evoked responses in all target electrodes, excluding the stimulation site, were obtained as 0.55 ± 0.20, 3.0 ± 0.87, and 6.0 ± 1.4 bit at 24, 45, and 70 DIV, respectively. The significance of the TE value was confirmed at 24–45 DIV (*p* = 1.0 × 10^−3^, Kruskal-Wallis test followed by Mann-Whitney *U* test). The TE values increased with the culture period, as in the case of spontaneous activity. In the evoked responses, the absolute TE values differed significantly from those of spontaneous activity. The reason for these differences is that the spatiotemporal patterns of spontaneous activity differ from those of evoked responses.

To confirm that the TE values are not biased by the firing rate increasing with culture days, TE values were calculated from surrogate datasets in which the temporal structure of the spike trains was randomized while preserving the original firing rates for measured spontaneous activity at 24, 45, and 70 DIV. The total TE values of the surrogate spontaneous activity in all target electrodes were obtained as 4.0 × 10^−4^ ± 9.37 × 10^−5^, 2.1 × 10^−3^ ± 5.5 × 10^−4^, and 3.8 × 10^−3^ ± 7.5 × 10^−4^ bit at 24, 45, and 70 DIV, respectively. The difference between spontaneous activity and surrogate spontaneous activity at 24, 45, and 70 DIV was significant (24 DIV, *p* = 1.3 × 10^−3^; 45 DIV, *p* = 5.0 × 10^−4^; 70 DIV, *p* = 8.2 × 10^−5^; Student’s *t* test). The TE values of the surrogate spontaneous activity were one to two orders of magnitude lower than those of the measured spontaneous activity. These findings indicate that TE values are not biased by an increase in the AWFR with network maturation. These results suggest that TE values reflect the strength of information flow between neurons, which changes with synaptic formation and network maturation processes.

**Figure 6:**
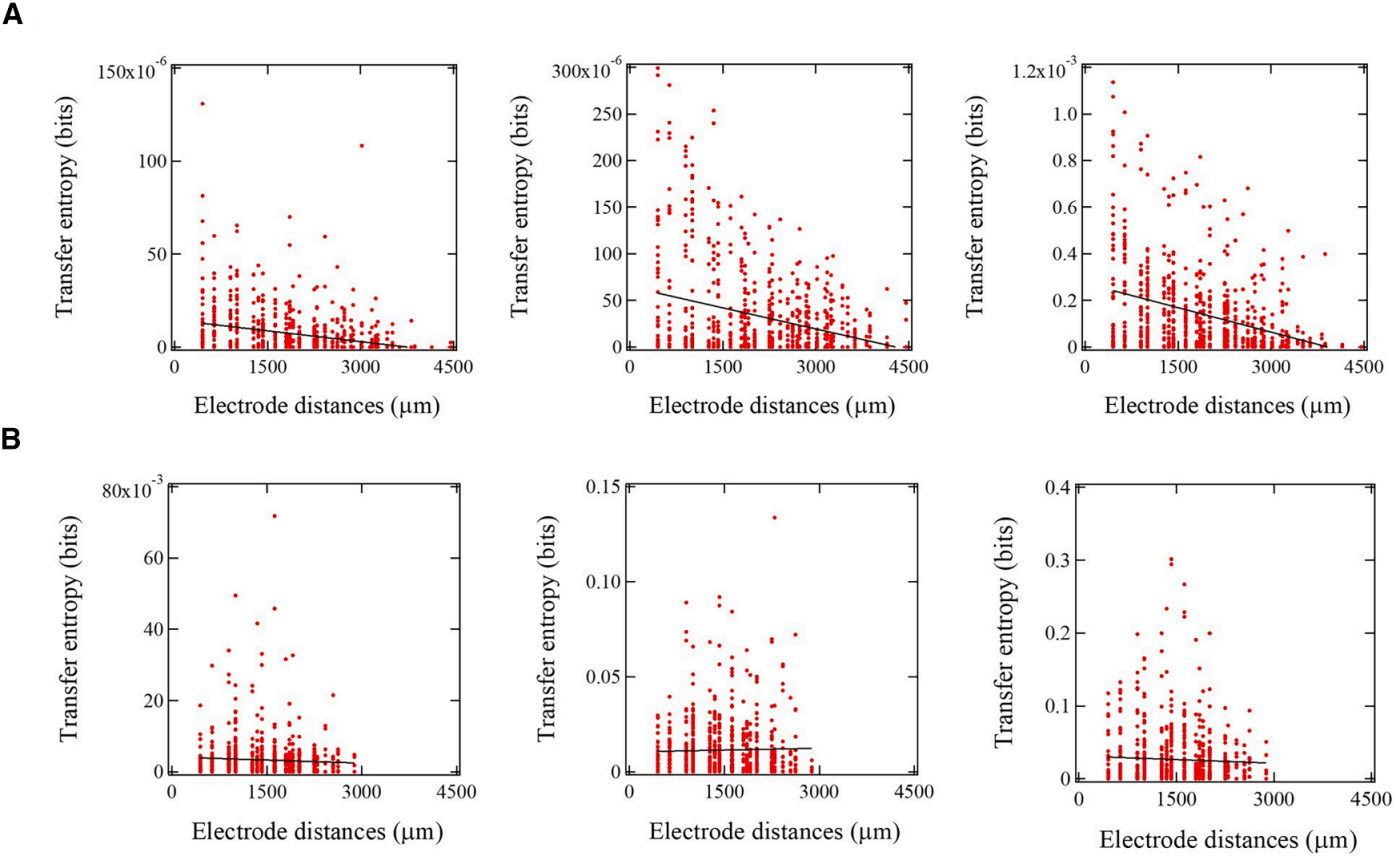
Scatterplots of TE values depending on electrode distance at 24 (left), 45 (middle), and 70 (right) DIV. (A) Total TE values for each electrode against the target electrode, with the maximum TE value for the spontaneous activity. (B) Total TE values in each target electrode against the electrode distance from the stimulation site in the stimulus-evoked responses.

If TE values indicate effective neuronal connectivity between neurons, TE values may depend on the distance between the microelectrodes. Therefore, as shown in Figure 6A, we plotted the TE values at the target electrode with the maximum TE against the electrode distances from the target electrode in the spontaneous activity at each culture stage. We evaluated the relationship between TE values and electrode distance using PCC. The PCC values at 24, 45, and 70 DIV were −0.27, −0.28, and −0.33, respectively. Despite the maturation of neuronal networks, TE values and electrode distances remained weakly negatively correlated, indicating that effective connectivity was not modified in neurons in the far area compared to those in the near area during network maturation.

The TE values for each target electrode against the electrode distance from the stimulation site at each culture stage are shown in Figure 6B. The PCC values derived from the total TE values were −5.4 × 10^−2^, 2.5 × 10^−2^, and −4.6 × 10^−2^ at 24, 45, and 70 DIV, respectively. In the evoked responses, there was a lower negative correlation than that of spontaneous activity because the electrical inputs induced a stable pattern in the whole neuronal network. These results suggest that evoked responses propagate in constant areas, leading to the repetition of stable neuronal electrical activity patterns.

### Relationship between spontaneous activity and evoked responses in TE analysis

**Figure 7:**
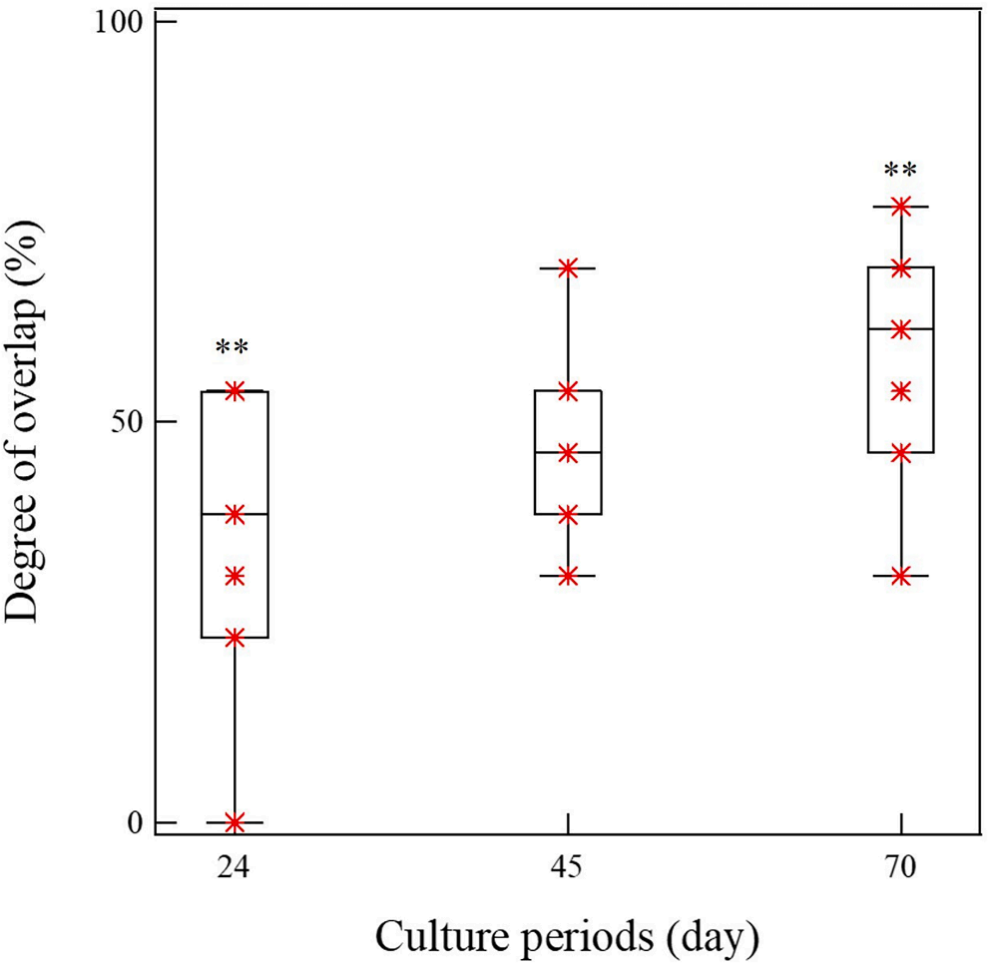
Relationship between spontaneous activity and evoked responses. Overlap probability of extracted target electrodes between spontaneous activity and evoked responses. ∗∗*p* < 0.01 indicates significant differences (Kruskal-Wallis test followed by Mann-Whitney *U* test).

To evaluate the connection characteristics in detail, neuronal electrical activity in target electrodes that input strong information flow (i.e., higher TE values) was analyzed for spontaneous activity and evoked responses. Several studies using *in vitro* and *in vivo* neuronal networks have shown that evoked responses and spontaneous activity are not independent and that some patterns of spontaneous activity are extracted by inputting electrical stimuli.^38^^,^^39^^,^^40^^,^^41^^,^^42^^,^^43^^,^^44^^,^^45^ If the TE values reflect the effective connectivity between neurons in neural networks cultured on MEAs, neurons with stronger inputs are considered similar in terms of spontaneous activity and evoked responses. In the TE matrix of spontaneous activity and evoked responses, the target electrodes with TE values in the 80th percentile (13 electrodes in a total of 64 electrodes) were extracted, and the degree of overlap between the selected 13 electrodes in spontaneous activity and evoked response points was analyzed at each recording point, as shown in Figure 7. The degrees of overlap between spontaneous activity and evoked response were 40.0% ± 12.3%, 47.6% ± 9.7%, and 58.2% ± 13.3% at 24, 45, and 70 DIV, respectively. The significance of these values was confirmed at 24–70 DIV (*p* = 6.4 × 10^−3^, Kruskal-Wallis test followed by Mann-Whitney *U* test).

The neuronal populations around the target electrodes overlapped by approximately 40%–60% at 24, 45, and 70 DIV in both spontaneous activity and evoked responses, and these values increased with culture days. These results suggest that spontaneous activity and evoked responses represent partially similar activity patterns, as shown in previous studies. These TE analysis results are consistent with those of previous studies.

### Graph theoretical analysis of functional connectivity via TE

Visualizing the functional connectivity of the neuronal network is crucial for elucidating its function. The TE values reflect the strength of information flow between neurons; therefore, they can be defined as the weight of functional connectivity between microelectrodes. To analyze network characteristics, each microelectrode of the MEA with functional connectivity was regarded as a node.

**Figure 8:**
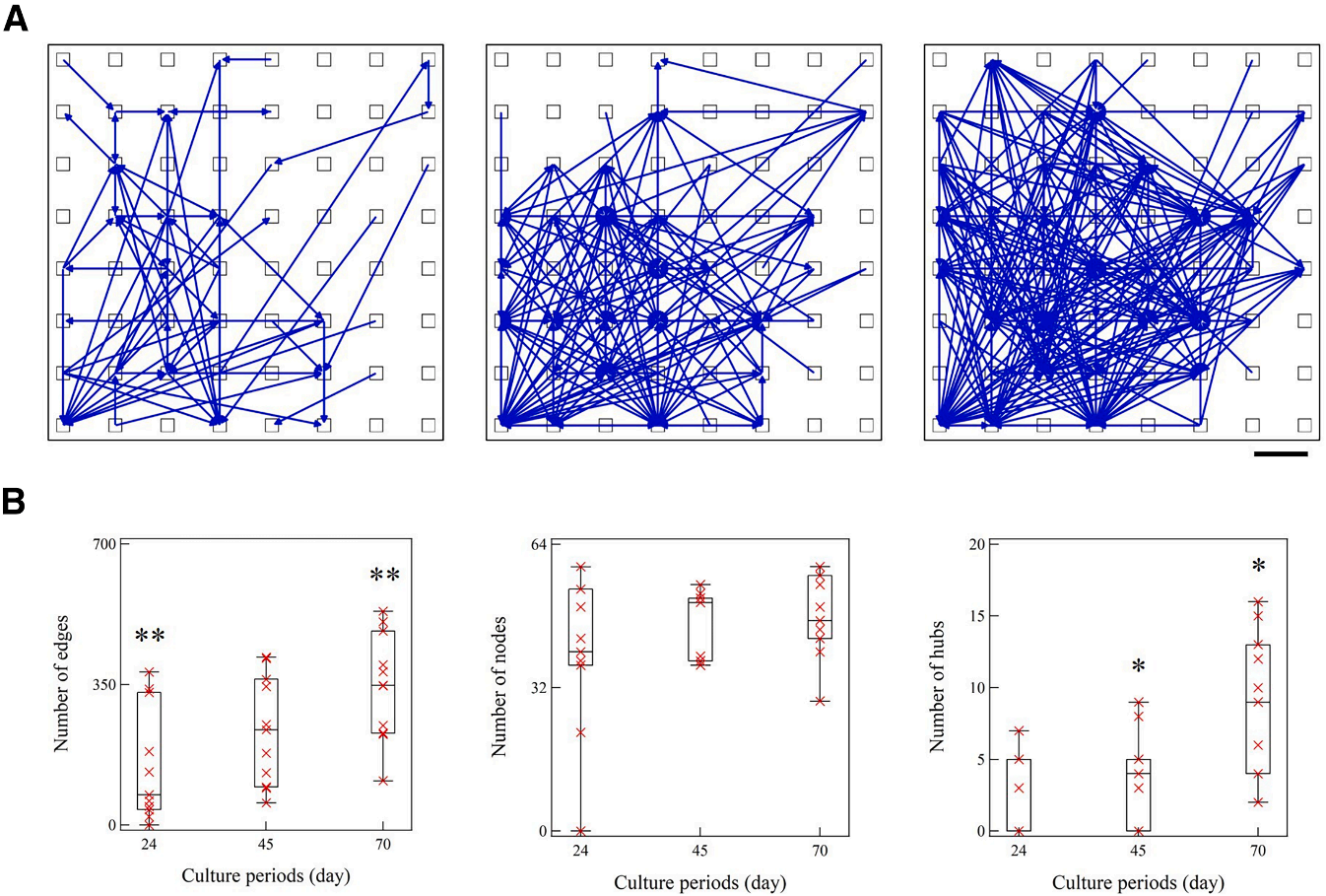
Graph theoretical analysis of functional connectivity of spontaneous activity based on TE. (A) Functional connectivity maps at 24 (left), 45 (middle), and 70 (right) DIV. The arrows indicate information transfer from the source to the target electrodes. (B) Number of edges (left), nodes (middle), and hubs (right) of cultured neuronal networks at 24, 45, and 70 DIV. ∗*p* < 0.05 and ∗∗*p* < 0.01 indicate significant differences (Kruskal-Wallis test followed by Mann-Whitney *U* test).

Next, a directed edge was drawn from the source to the target node pairs whose TE values exceeded the threshold defined by the mean plus the standard deviation of the entire TE matrix. Thus, a functional connectivity map was created for spontaneous activity measured by the MEA, as shown in Figure 8. The number of edges, nodes, and hubs of graph theory in cultured neuronal networks was evaluated using functional connectivity maps. The number of edges in neuronal networks was 145.9 ± 40.5, 234.5 ± 38.7, and 346.4 ± 38.2 at 24, 45, and 70 DIV, respectively. The significance of these values was confirmed at 24–70 DIV (*p* = 3.9 × 10^−3^, Kruskal-Wallis test followed by Mann-Whitney *U* test). The number of nodes in the neuronal networks was 39.9 ± 5.0, 45.9 ± 2.2, and 48.1 ± 2.6 at 24, 45, and 70 DIV, respectively. The significance of these values was not confirmed for each culture period (*p* = 0.39, Kruskal-Wallis test). The number of hubs in the neuronal networks was 1.8 ± 0.77, 3.5 ± 0.94, and 8.9 ± 1.4 at 24, 45, and 70 DIV, respectively. The significance of these values was confirmed at 45–70 DIV (*p* = 1.3 × 10^−2^, Kruskal-Wallis test followed by Mann-Whitney *U* test). These results indicate that both the number of edges and hubs of neuronal networks significantly increased over 70 days in culture. Several previous studies using cultured neuronal networks have reported that the number of edges and hubs increases over approximately 30–40 DIV.^46^^,^^47^ These findings obtained from the TE-based graph theoretical analysis revealed that information flow was strengthened after the maturation of neuronal networks.

## Discussion

In this study, we estimated the information flow strength between neurons by evaluating the causal relationships between microelectrodes in MED probes with TE values. In previous studies, CC has been generally used to estimate the effective connectivity between neurons.^7^^,^^13^^,^^14^ However, CC is a symmetrical analysis; therefore, the direction of the information flow between neurons on electrodes cannot be evaluated. In addition, CC has been reported to have high performance in linear neuronal circuit models composed of excitatory synaptic inputs but shows low performance in nonlinear models composed of excitatory and inhibitory synaptic inputs.^29^ Hippocampal neuronal networks are composed of both excitatory and inhibitory synaptic inputs; therefore, it is effective to use TE instead of CC in hippocampal neuronal networks. In the CC-based synchrony analysis, no significant changes were detected over 70 days in the cultures (Figure S2). These results indicate that CC analysis is insufficient to characterize neuronal dynamics during long-term cultivation because it linearly scans time lags to detect peaks reflecting spike-time overlaps. In contrast to CC analysis, TE analysis strongly shows the probability of specific spike-pattern occurrences across two time series, thus detecting the nonlinear dependencies between neurons. Compared to existing methods, TE analysis enables us to reveal the strength of information flow over 70 days in culture. The results showed that information flow strength increased with the culture period (Figure 5), and a weak correlation between the information flow strength and cellular distance was observed (Figure 6). These data suggest that TE values reflect effective neuronal connectivity driven by shared inputs, population coupling, and higher-order interactions between multiple neurons.

Previous studies have reported that both firing rates and burst frequencies of spontaneous activity in cultured neuronal networks saturate around 20–30 DIV.^10^^,^^11^^,^^48^^,^^49^ It has been shown that cultured neuronal networks acquired small-world properties after 14 DIV and that network topology indices saturated at 28 DIV,^46^ while network edge density stabilized between 12 and 30 DIV.^47^ These findings revealed that the functional maturation of neuronal networks was defined at approximately 30 DIV by conventional analysis. In contrast, our TE analysis supports that the information flow strength continued to increase between 45 and 70 DIV, indicating the developmental enhancement of functional connectivity over 70 days in cultured neuronal networks. In addition, we evaluated the graph theoretical network topology indices from the TE matrices (Figure 8). Notably, both the number of edges and hubs in cultured neuronal networks increased over 70 days in culture, suggesting that network topology transitions and computational capacity of neuronal networks continue to improve over 70 days of developmental periods. Thus, the findings of this study open perspectives on information flow in neuronal networks and provide novel biological insights into self-organization properties beyond existing network maturation during the continued development of neuronal networks.

The spatiotemporal electrical activity patterns of neuronal networks are formed according to the spatial propagation of spikes; therefore, novel methods for the spatial pattern propagation of spikes are required. TE analysis is considered to determine not only spike propagation in spontaneous activity but also that of evoked spikes induced by electrical inputs^14^^,^^31^ or optogenetic stimulation,^35^^,^^50^^,^^51^^,^^52^ suggesting that the functional correlation between evoked responses and spontaneous activity can be analyzed using TE. Spike patterns in neuronal modules that form assembled neurons to reconstruct brain structures have been reported in several studies.^35^^,^^53^^,^^54^ The TE analysis suggested in this study can also be used to evaluate spatial spike propagation between neuronal modules. Because the number of channels is limited in conventional MEAs, including MED probes, the spatial resolution is insufficient to measure neuronal electrical activity at the single-neuron level. The measurement of extracellular potentials from several thousand points simultaneously with a complementary metal-oxide semiconductor (CMOS)-based high-density MEA (HD-MEA) system has been developed,^55^^,^^56^^,^^57^^,^^58^ and TE analysis is also useful for evaluating the information flow strength between single neurons using HD-MEA-based electrical measurements.

In this study, we did not employ generalized TE^59^ and local TE^60^ to analyze neuronal dynamics, including single spikes, network bursts, and quiescent periods over a time range as long as 10 min. Generalized TE can separate states, such as burst and nonburst periods, and local TE can detect information-rich events, such as network bursts, by calculating TE with a tiny temporal resolution. These approaches, based on the temporal separation of measured spike datasets, are not suitable for analyzing long-term spontaneous activity that continues to alter in various states in this study. The TE analysis employed in this study used the same lag parameter for both single spikes and network burst periods in the 10 min spike datasets. This method has limitations for highly accurate extraction of neuronal dynamics. To address this limitation, employing multiscale TE^61^, which analyzes TE with multiple lag parameters, may achieve a more accurate extraction of neuronal dynamics. Therefore, multiscale TE has the potential to provide a more reliable conclusion for neuronal dynamics.

## Conclusion

TE values were used to evaluate the information flow strength between neurons from spike trains measured using MEAs. We used MEAs with 64 microelectrodes in an array with 450 μm spacing to measure spontaneous activity and evoked responses over 70 days. The TE values used as an index of information flow strength were calculated for all electrode pairs, and the TE values of spontaneous activity and evoked responses increased with the culture period. These results suggest that maturation of the neural network facilitates information flow strength between neurons. Moreover, we showed the relationship between information flow strength and cellular distance, and a weak correlation was observed. We found that 40%–60% of the electrodes with strong input were shared between spontaneous activity and evoked responses. These results strongly support the notion that spontaneous activity and evoked responses are not individual responses but that a part of the spontaneous activity pattern is extracted by electrical stimuli in evoked responses. We performed a TE-based graph theoretical analysis of functional connectivity, which revealed that both the number of edges and hubs of neuronal networks increased with the culture period. These results suggest that the network topology continued to transition for 70 days in culture, which was longer than the maturation period observed in conventional analysis methods. Therefore, TE analysis is an effective tool for elucidating the properties of neuronal electrical activity.

## Data and code availability

The data that support the findings of this study are available from the corresponding author upon reasonable request.

## Acknowledgments

This research was supported by the 10.13039/501100001691JSPS KAKENHI Grant-in-Aid (grant nos. JP 25K21349 for W.M. and JP22H05140, JP23K18511, and JP23H03501 for C.H.) and by the JST FOREST Program (grant no. JPMJFR2053 for C.H.).

## Author contributions

Conceptualization, W.M., Y.S., and C.H.; methodology, W.M., Y.S., and C.H.; software, W.M.; validation, W.M., Y.S., and C.H.; formal analysis, W.M. and Y.S.; investigation, W.M., Y.S., and C.H.; data curation, W.M. and Y.S.; writing, W.M., Y.S., and C.H.; supervision, W.M. and C.H.; project administration, W.M. and C.H.; funding acquisition, W.M. and C.H. All authors have read and agreed to the published version of the manuscript.

## Declaration of interests

The authors declare no competing interests.

## Supplemental information

Document S1. Figures S1 and S2 and supplemental materials and methods

Document S2. Article plus supplemental information
