# Supplementary material for: Information flow in cultured neuronal networks by transfer entropy
Source: Biophys Rep (N Y). 2026 Jul 20;6(3):100278. doi: 10.1016/j.bpr.2026.100278 (PMC13476481; doi:10.1016/j.bpr.2026.100278)
Supplement: Supplementary file 1 [file mmc1.pdf]

Biophysical Reports, Volume *6*

Supplemental information

Information flow in cultured neuronal networks by transfer entropy

Wataru Minoshima, Yumi Segawa, and Chie Hosokawa

Cross-correlation analysis

To evaluate neuronal synchrony, cross-correlation (CC) analysis was performed based on our previous report with minor modifications (1). In brief, the CC value $C_{xy}(\tau)$ between pairs of electrodes x and y is calculated as follows:

$$C_{xy}(\tau) = \frac{1}{\sqrt{N_x N_y}} \sum_{t_i=\tau-\frac{\Delta\tau}{2}}^{\tau+\frac{\Delta\tau}{2}} X(t_s)Y(t_s - t_i) \quad (1)$$

where N_x and N_y are the total numbers of spikes in electrodes x and y, respectively. τ is the time lag, $\Delta\tau$ is the time bin of 2 ms, and t_s and t_i are the spike timestamps at the current time s and delay time i, respectively. The coincidence index (CI) is calculated to quantify the synchrony between pairs of electrodes. The CI value is the ratio of the summation of cross-correlation values in ±5 ms around the correlation peak at τ = 0 to the summation of those values in ±150 ms around the correlation peak and is calculated as follows:

$$\mathrm{CI} = \frac{\sum_{\tau=-k}^{k} C_{xy}(\tau)}{\sum_{\tau=-T}^{T} C_{xy}(\tau)} \quad (2)$$

where k and T are the number of bins around the correlation peak, which are set to 5 and 150, respectively. CC analysis was performed using a custom script based on Python 3.13.

Statistics

A pairwise Student's t-test was used to compare the summation of TE values between bin widths of 1 and 5 ms. Significance was defined when p-values were less than 0.05. All statistical tests were performed using a custom-made script based on Python 3.13.

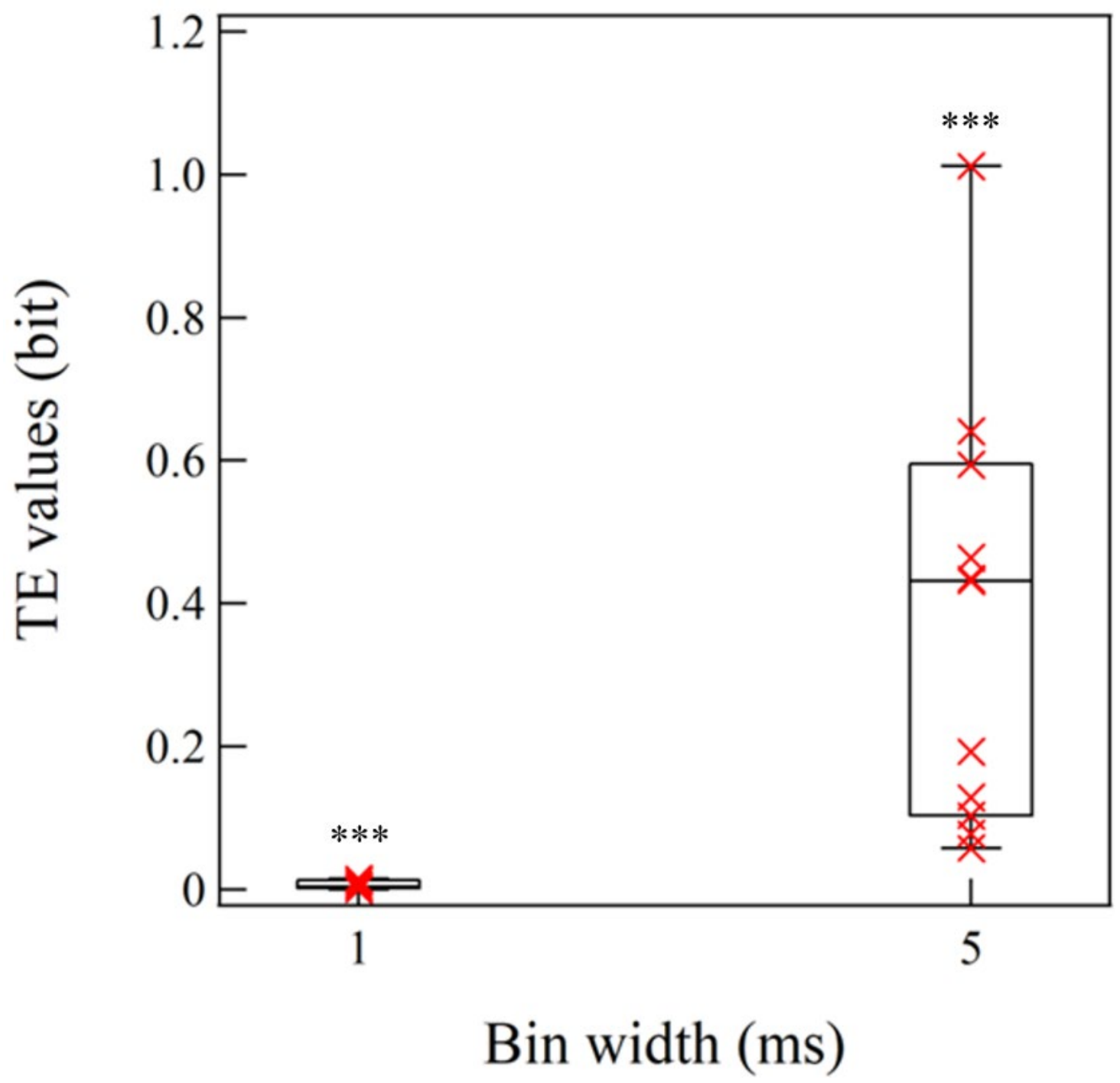

Figure S1 Bin width dependence of the TE values in the TE analysis. Summation of TE values of spontaneous activity with bin widths of 1 and 5 ms. *** indicates significant differences (***, $p < 0.001$; Student's *t*-test).

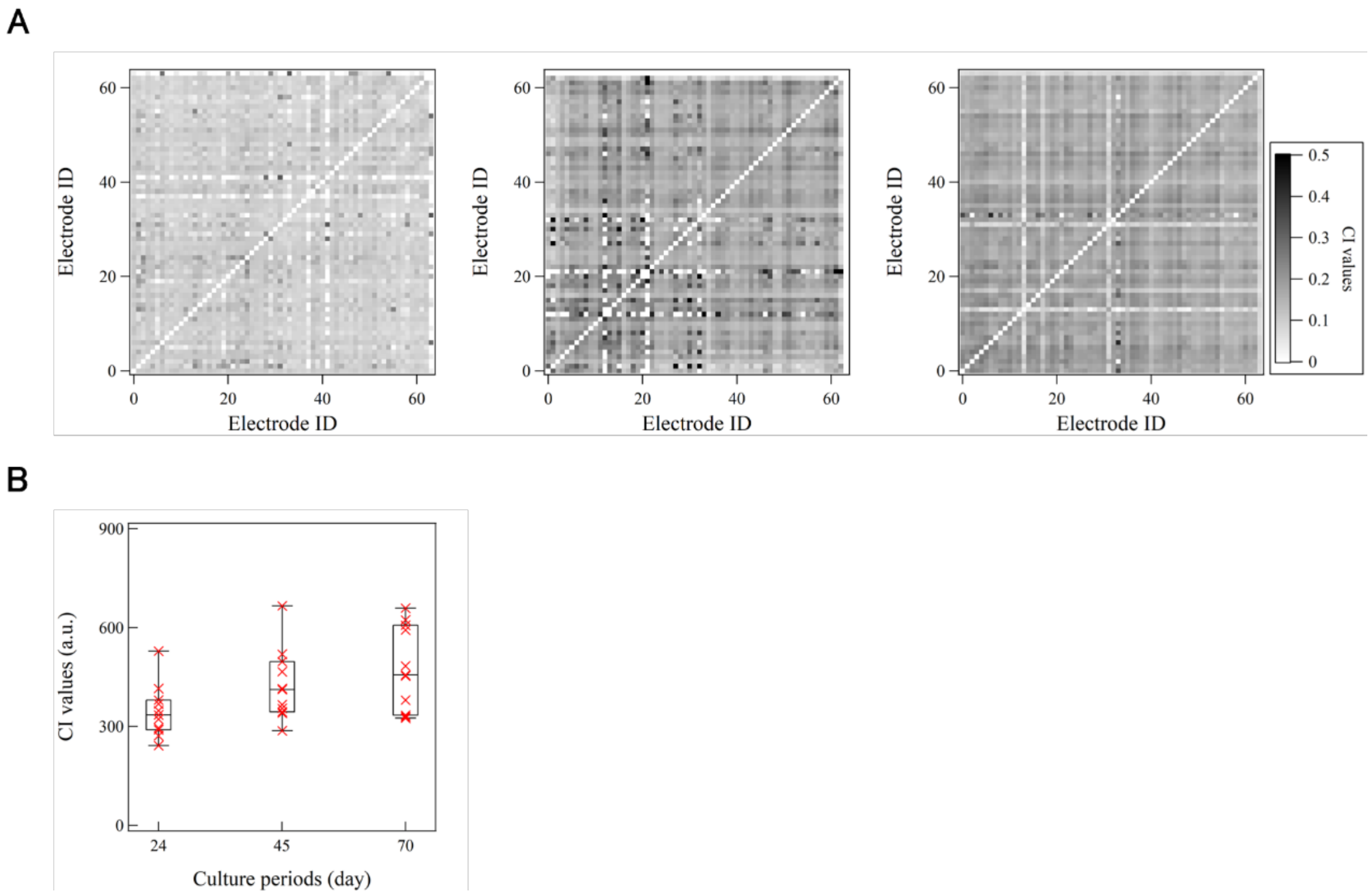

Figure S2 Cross-correlation analysis of spontaneous activity. (*A*) Matrices of CI values in spontaneous activity at 24 (left), 45 (center), and 70 (right) DIV. (*B*) Summation of the CI values in the spontaneous activity at all pairs of electrodes at each culture stage.

Supporting references

(1) Segawa, Y., W. Minoshima, K. Masui, and C. Hosokawa. 2026. Spatiotemporal patterns of neuronal electrical activity evoked by stimulation with a focused femtosecond laser. *Biochem. Biophys. Res. Commun*. 794:153049.
